# Region and cell-type resolved quantitative proteomic map of the human heart

**DOI:** 10.1038/s41467-017-01747-2

**Published:** 2017-11-13

**Authors:** Sophia Doll, Martina Dreßen, Philipp E. Geyer, Daniel N. Itzhak, Christian Braun, Stefanie A. Doppler, Florian Meier, Marcus-Andre Deutsch, Harald Lahm, Rüdiger Lange, Markus Krane, Matthias Mann

**Affiliations:** 1 Department of Proteomics and Signal Transduction, Max Planck Institute of Biochemistry, Martinsried, 82152 Germany; 2 Novo Nordisk Foundation Center for Protein Research, Faculty of Health Sciences, University of Copenhagen, Copenhagen, 2200 Denmark; 3 Department of Cardiovascular Surgery, German Heart Center Munich at the Technische Universität München, Munich, 80636 Germany; 4 Forensic Institute, Ludwig-Maximilians-University, Munich, 80336 Germany; 5 DZHK (German Center for Cardiovascular Research), Partner Site Munich Heart Alliance, Munich, 80802 Germany

## Abstract

The heart is a central human organ and its diseases are the leading cause of death worldwide, but an in-depth knowledge of the identity and quantity of its constituent proteins is still lacking. Here, we determine the healthy human heart proteome by measuring 16 anatomical regions and three major cardiac cell types by high-resolution mass spectrometry-based proteomics. From low microgram sample amounts, we quantify over 10,700 proteins in this high dynamic range tissue. We combine copy numbers per cell with protein organellar assignments to build a model of the heart proteome at the subcellular level. Analysis of cardiac fibroblasts identifies cellular receptors as potential cell surface markers. Application of our heart map to atrial fibrillation reveals individually distinct mitochondrial dysfunctions. The heart map is available at maxqb.biochem.mpg.de as a resource for future analyses of normal heart function and disease.

## Abstract (web summary)

The human heart is composed of distinct regions and cell types, but relatively little is known about their specific protein composition. Here, the authors present a region- and cell type-specific proteomic map of the healthy human heart, revealing functional differences and potential cell type markers.

## Introduction

The human heart beats more than two billion times in an average life span and each contraction is precisely controlled by an intricate interplay between electrical signals and mechanical forces. At the anatomical level, it is composed of four cavities, four valves, large arteries, and veins, which act in concert to achieve proper filling, ejection, contraction, and overall pump function. The heart’s own blood supply is ensured by two coronary arteries. The human heart is composed of four major cell types—cardiac fibroblasts (CFs), cardiomyocytes, smooth muscle cells (SMCs), and endothelial cells (ECs)^1^. Their proportion with respect to number and volume, however, remains controversial. CFs are mesenchymal cells, which produce the extracellular matrix (ECM) scaffold of the heart and are thought to constitute more than half of all heart cells^2^. Cardiomyocytes are estimated to provide about 30% of the total cell number but account for over 70% of the total cardiac mass because of their large volume. In contrast, SMCs, which support the vascular system, and ECs, which form the interior lining of the heart, blood vessels, and cardiac valves, are generally believed to be much less abundant. However, these estimates have been challenged and a recent report claims that ECs are the largest cellular population within the heart^3^.

In common with other muscle tissues, the heart is dominated by a small number of proteins involved in the contractile apparatus. It employs tissue-specific isoforms such as cardiac troponins, which are used in the diagnosis of myocardial infarction. From a physiological and pathophysiological perspective, it would be desirable to gain deeper insights into the molecular characteristics of the heart at the spatial and cellular levels. In particular, characterization of the healthy state of the human heart would be an important starting point to investigate heart disease, which—despite major progress remains the leading cause of death in developed countries and is rapidly increasing in developing ones^4^.

Relatively little is known about the protein composition of the different regions and cell types of the heart. Previous studies have focused on defining differences between specific regions of the heart, or single-diseased heart compartments^5^, or from nonhuman, or subcellular material^6,7^. Phosphoproteomic studies have also been applied for the analysis of mammalian hearts^8,9^. Moreover, other studies use transcriptomic approaches^10–12^, which is an imperfect proxy for protein levels and their dynamics. However, proteins are the driving forces of the cellular machinery and they are involved in the control of virtually all physiologic events. The high dynamic range of the muscle proteome presents a formidable challenge to the comprehensive analysis of the heart at the level of expressed proteins. This is because very abundant proteins make it difficult to detect low abundant regulatory proteins in the same sample. The majority of studies only identified a few thousand proteins, and there is a paucity of studies of the human, nondiseased heart, because of the difficulty in obtaining the relevant tissue.

A global protein expression “footprint” of the healthy heart can be used as a reference library to compare against footprints of malfunctioning hearts in the search for biomarkers, therapeutic targets, or disease signatures. Recent advances in MS-based proteomics technology now allow the identification of very deep proteomes^13,14^. Our group has already established proteomics maps of the mouse liver and brain^15,16^ and analyzed skeletal muscle in considerable depth and sensitivity^17,18^. Here, we set out to generate a spatial and cell-type-resolved proteomic map of the healthy human heart. To this end, we measured 16 regions of three human hearts, as well as primary cell types. We employed high-sensitivity sample preparation, peptide fractionation, and an advanced label-free LC–MS workflow to quantify a total of more than 11,000 proteins. Our results establish proteomic differences between heart regions, suggest functional differences, and pinpoint potential cell-type markers. To illustrate the usefulness of the heart proteomic map, we apply it to define molecular changes in patients suffering from atrial fibrillation (AFib).

## Results

### Establishing a proteomic map of the human heart

**Fig. 1:**
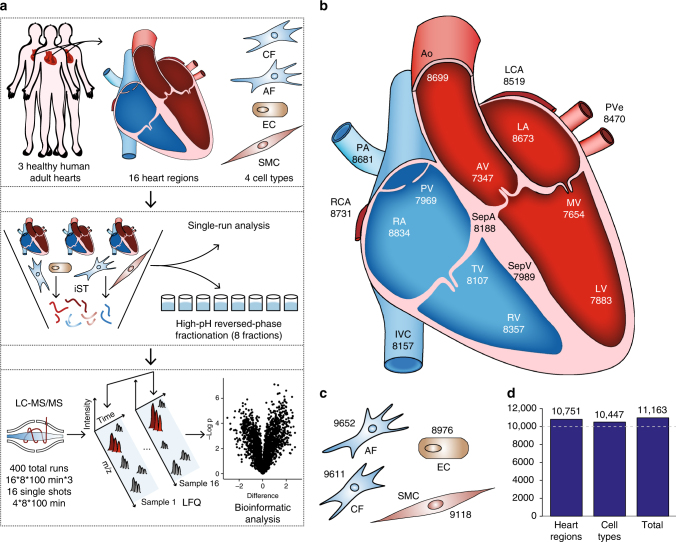
The quantitative landscape of the human heart proteome. **a** Experimental design, including the source of material (upper panel), in-depth vs. single-run analyses (middle panel), and schematic depiction of the analytical workflow (lower panel). **b** Graphical illustration of the human heart showing the total number of quantified proteins in each region. **c** Quantified proteins in three cardiac cell types and adipose fibroblasts. **d** Bar plot of the total number of quantified proteins in all heart regions, cell types, and the entire data set

Three adult hearts were obtained from male trauma victims aged 21–47 years with no apparent adverse heart condition (Supplementary Table 1). We selected a total of 16 anatomically defined regions from each heart for MS analysis (Fig. 1a, b): the atrial and ventricular septa (SepA and SepV) separating the atria and ventricles, respectively; the right atrium (RA) and right ventricle (RV) connected via the tricuspid valve (TV); the left atrium (LA) and left ventricle (LV) linked via the mitral valve (MV); the right and left ventricles connected to the pulmonary artery (PA) and aorta (Ao) via the pulmonary and aortic valves (PV and AV); the inferior vena cava (IVC) collecting deoxygenated blood; the pulmonary vein (PVe) carrying oxygenated blood; and the main right and left coronary arteries (RCA and LCA) supplying the heart with oxygen-rich blood. In addition, we isolated CFs, ECs, and SMCs from patients undergoing cardiovascular surgery (Fig. 1c).

After tissue homogenization in liquid nitrogen, we performed all sample preparation using the “in-StageTip (iST) method” (see “Methods” section), reducing sample contamination, loss, preparation time, and increasing quantification accuracy^19^. The recently described “loss-less” nano-fractionator enabled efficient fractionation of a total of only 30 µg of peptides into eight fractions, of which a third of each fraction was loaded in the subsequent LC–MS step^20^. The resulting 400 samples were analyzed with a state-of-the-art label-free workflow on a quadrupole– Orbitrap mass spectrometer (Fig. 1a).

Analysis in the MaxQuant environment using a false-discovery rate (FDR) of less than 1% at the peptide and protein levels^21^, identified a total of 181,814 sequence-unique peptides. These assembled into 11,236 protein groups. Many high-abundance proteins had very high sequence coverage—such as 100% for myosin regulatory light chain 2 (MYL7)—whereas median coverage of all proteins was ~38%. The MaxLFQ algorithm^22^ quantified 11,163 proteins, 10,751 in the 16 heart regions, and 10,447 in the noncardiomyocyte cell types, including AFs (Fig. 1d). Proteomic depth was high in all regions, including the four cardiac valves, in which we identified a mean of about 7800 proteins despite the fact that it mainly consists of ECM. To put this number in perspective, reanalysis of the “human draft proteome” heart data^23^ with the settings used here revealed that our study identified more than three times as many proteins, most of which were of low abundance (Supplementary Fig. 1). For further analysis, we only considered a subset of 8908 proteins with quantitative values in all biological triplicates of at least one heart region.

Signal intensities for the quantified proteins spanned more than six orders of magnitude, while only six proteins—myosin 7 (MYH7), titin (TTN), cardiac muscle-specific actin (ACTC1), alpha-actinin-2 (ACTN2), and hemoglobin (HBA1 and HBB) represented 25% of the total protein molecules in cavities, with similar values in vessels and valves (Supplementary Fig. 2a–f). A large amount of hemoglobin remained, despite extensive washing of the samples with PBS, since hearts of trauma victims cannot be perfused. Due to efficient peptide fractionation, our measurement covered regulatory proteins such as transcription factors GATA4, GATA6, TBX20, TBX3, and TBX5 controlling cardiac-specific gene expression (Supplementary Fig. 3).

To assess quantitative reproducibility, we analyzed several samples in technical triplicates. Pearson correlation coefficients (0.97–0.99) were on par with, or exceeded the values previously achieved in cell line systems^24^ (Supplementary Fig. 4). Likewise, we observed high correlation values between biological replicates; ranging from 0.83 (PVe) to 0.95 (LA) (Supplementary Fig. 5). As these values incorporate any differences due to postmortem sample treatment, we conclude that our results from three individuals can likely be generalized to the adult male population at large. Raw data and MaxQuant results are provided online and the human cardiac proteome resource is available in our online database MaxQB^25^ (see below).

### Comparative analysis between anatomical areas of the heart

**Fig. 2:**
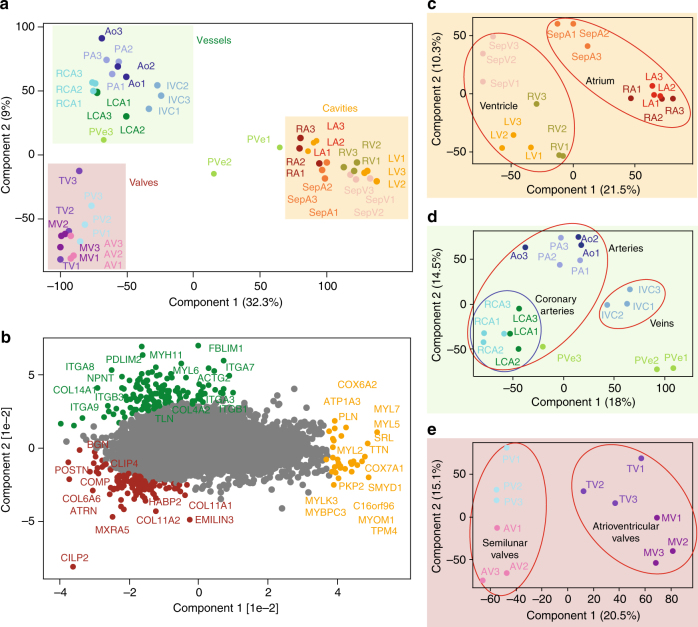
Principal component analysis (PCA) of the 16 heart regions based on their proteomic expression profiles. **a** The proteomes of the cavities (RA, LA, RV, LV, SepA, and SepV), vessels (Ao, PA, RCA, LCA, IVC, and PVe), and valves (TV, MV, AV, and PV) depicted by replicate number (individuals 1, 2, and 3). The first and second component segregate the heart areas and account for 32.3 and 9% of the variability, respectively. **b** Proteins driving the segregation between the three heart areas. **c** Cavities segregate into the ventricular and atrial part, **d** vessels into coronary arteries (RCA, LCA) and outgoing vessels (Ao, PA), and **e** valves into ventricular (MV, TV) and semilunar valves (AV, PV)

For an overall assessment of proteomics similarities and differences of the 16 heart regions, we employed principal component analysis (PCA). Cavities, vessels, and valves clearly clustered separately with the samples from different individuals tightly grouped together (Fig. 2a). The only exception was the PVe from patient 1 and 2, which clustered closer to the atrium than the other vessels. This is readily explained by the difficulty of resecting PVe without contamination from LA; thus, PVe samples were excluded from the subsequent analyses.

The segregation of the three groups was mainly driven by MYL7, MYL5, cytochrome c oxidase subunit 7A1 (COX7A1), sarcalumenin (SRL) and TTN (highlighted in red, driving segregation of the cavities), and collagen proteins, such as COL4A2, COL14A1, and integrins, including ITGA7, ITGA8, and ITGB1 (highlighted in blue, segregating vessels), as well as biglycan (BGN), COL11A1, COL11A2, and COL6A6 (highlighted in purple, segregating valves) (Fig. 2b). As these proteins reflect known biological differences between the cardiac cavities and the vessels and valves that are rich in ECM components, they serve as positive controls of our proteomic analysis. Furthermore, the PCA analysis highlighted several interesting candidates, such as cartilage intermediate layer protein 2 (CILP2) and MXRA5 (valves), nephronectin (NPNT), a functional ligand of ITGA8 and ITGB1 (vessels), and uncharacterized proteins such as C16orf96 (cavities).

Each of the three main clusters exhibited further subgroupings. Heart cavities were divided into atrial (RA, LA, and SepA) and ventricular (RV, LV, and SepV) parts and within them atrial and ventricular septa were separated from atria and ventricles, respectively. Furthermore, there was a moderate but clear distinction of the left and right side of the heart (Fig. 2c). The vessel group subdivided into large arteries (Ao and PA) and large veins (IVC) (Fig. 2d). Within the arteries, the RCA and LCA formed a subcluster, demonstrating differences between coronary and large arteries at the proteomic level. Finally, both atrioventricular valves (MV and TV) clustered together, whereas semilunar valves (AV and PV) formed a separate group (Fig. 2e). The main drivers of the PCA separation are highlighted in Supplementary Fig. 6.

**Fig. 3:**
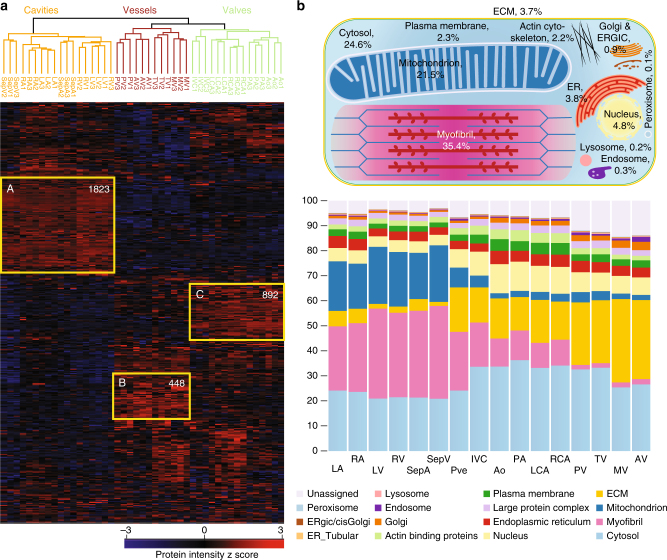
Proteins differentially expressed across the different heart areas. **a** Heat map of z-scored protein abundances (LFQ intensities) of the differentially expressed proteins (ANOVA, FDR < 0.05) after unsupervised hierarchical clustering reveals proteins significantly upregulated in the cavities, vessels, or valves (highlighted in yellow: A, B, and C). **b** The upper panel shows a schematic of an average heart cavity cell, where organelles are sized according to their contribution to total protein mass. Percentages are taken from the median of all cavities and scaled to account for unassigned proteins. The lower panel shows the contribution of each organelle to cellular protein mass, as a percentage of the total, in each heart region

For a functional view of the proteomic differences in the human heart, we performed unsupervised hierarchical clustering of the 6807 proteins with statistically different expression across the heart regions (FDR < 0.05) (Supplementary Data 1). This again clustered individuals in all but one case (RV of one individual), followed by cavities, vessels, and valves with their subdivisions (Fig. 3a). The heat map shows one major cluster of highly and coexpressed proteins for each of the three anatomical areas. Gene ontology and GSEA^26^ revealed that proteins in cluster A (high expression in the cavities) were enriched (*p* < 10^−12^) in terms of cardiac muscle contraction, Z disc, and sarcomere organization compared to clusters B and C (high expression in the vessels and valves, respectively). The terms mitochondria and respiratory electron transport chain were also enriched (*p* < 10^−55^) in this cluster, concordant with the large number of mitochondria to ensure sufficient amounts of ATP for continuous muscle contraction (Supplementary Data 2). Thus, our proteomic data provide a global protein expression basis for the functional specialization of cardiac muscle tissue.

To provide insights into the organelle sizes in the heart proteome at a quantitative level, we used the proteomic ruler approach to estimate copy numbers per cell^27^ together with subcellular localization annotations^28,29^. We calculated an approximate total protein content of 1 ng per diploid nucleus and found that a heart cavity muscle cell has an approximate volume of 5 pL per nucleus (note that about 30% of all cardiomyocytes have two or more nuclei^2^). These values are roughly double that of mouse heart muscle cells^30^. Estimated protein copy numbers per diploid nucleus and protein concentration^31^ across our samples ranged from ~10 to 10^9^ and <0.1 nM to 200 µM, respectively (Supplementary Data 3). We found that mitochondria constituted 21% of protein mass in the cavities (Fig. 3b, “Methods” section, and Supplementary Data 3). This compares to 7% of mitochondrial protein mass in HeLa cells^28^ and 3% in valves and vessels, demonstrating the immense aerobic respiration in cardiac muscle cells localized in the atrial and ventricular part of the heart at a quantitative level.

To further mine our quantitative and in-depth proteome resource, we used volcano plots^32^ to compare expression differences within the three anatomical areas of the heart. We specifically focused on proteins that were in the top 75% in abundance and only identified in one of the two regions that we compared (Supplementary Data 4).

### The atrial vs. ventricular proteome

**Fig. 4:**
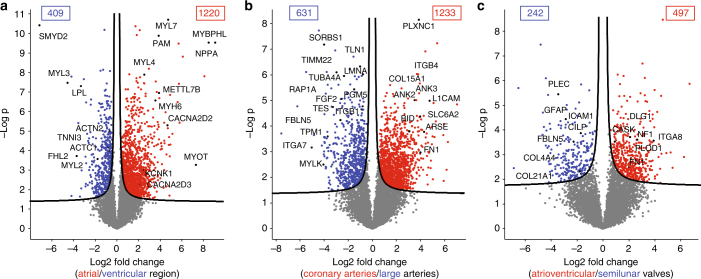
Proteins differentially expressed in human heart regions. Volcano plots of the *p* values vs. the log2 protein abundance differences between regions, with proteins outside the significance lines colored in red or blue (FDR < 0.05). *p* values are calculated from the data of three healthy hearts. **a** Ventricular (LV, RV, and SepV) compared to the atrial (LA, RA, and SepA) regions, **b** coronary arteries (LCA, RCA) compared to arteries (Ao, PA), and **c** semilunar (AV, PV) compared to atrioventricular (TV, MV) valves

Although they are both heart muscles, the main role of atria is to collect and transfer pulmonary and systemic blood, whereas ventricles need to pump the blood throughout the entire body. Consistent with these different functions, we found drastic differences in their proteomes, with 1220 (13.7%) proteins showing significantly higher expression in the atria and 409 (4.6%) displaying higher expression in the ventricles (Fig. 4a). As expected, mitochondrial proteins were more abundant in ventricles (*p* < 10^−150^ by GSEA analysis, Supplementary Data 5). Cardiomyocytes use fatty acids as their main energy source and ventricular myocytes have higher energy demands due to the greater force of contraction. Accordingly, lipid metabolic processes were overrepresented in the ventricular region (*p* 7 × 10^−26^), exemplified by the ~tenfold increased expression of lipoprotein lipase (LPL). Likewise, it was enriched in muscle contraction (*p* 9 × 10^−27^), due to increased cardiomyocyte size, as estimated by the proteomic ruler (“Methods” section). The known markers for ventricles vs. atria, such as MYL2, MYL3, and LPL were clearly recovered as such, and our data set contains many additional ones, including the lysine methyltransferase SMYD2, which is thought to have a role in myocyte function^33^. Several interesting candidates were only identified in the ventricles but not in the atria, such as the probable histone demethylase JMJD1C, the ubiquitin ligase TRIM38, the tumor suppressor RASSF8, and the uncharacterized KIAA1324L protein. These proteins have not been associated with ventricular functions before and suggest starting points for exploring their role in heart physiology. Proteins previously reported as atrium specific^34–36^, including myosin 6 (MYH6), peptidyl-glycine alpha-amidating monooxygenase (PAM), and natriuretic peptides A (NPPA) displayed ten to several hundred-fold higher abundance in the atrium. Interestingly, these proteins are only highly expressed in the ventricular regions under pathological conditions—for example, cardiac hypertrophy leads to elevated PAM levels in ventricles^37^. We found potassium ion channels predominantly in the atrial part, such as KCNK1, which induces background currents^38^, and calcium-dependent ion channels, including CACNA2D2 and 3 (>fourfold), as well as gap junction GJA5 (>ninefold), reflecting the presence of the sinus and AV node, which generate the electrical impulse for heart contraction. Others, such as CACNA1C, which play an important role in excitation–contraction coupling in the heart, were equally expressed in the atria and ventricles (see also Supplementary Fig. 7).

The high protein sequence coverage encouraged us to investigate isoform-specific expression patterns of sarcomeric proteins (Supplementary Data 6). These isoforms are of particular importance because their altered expression has been associated with diverse cardiac dysfunctions^39^ and because troponins are routinely used biomarkers for myocardial infarction^40^. The myosin isoform families MYH6, MYH7, MYL2-7, and MYL8 localized highly specifically to atria or ventricles, confirming the regional specificity of our data set despite high sequence identity, for instance, over 80% between MYHs^41^.

Myocardial infarction remains one of the largest causes of death and although rapid ELISA tests against cardiac troponins TNNT2 and TNNI3 play a crucial role, further improvements in diagnosis would be of great clinical benefit^42,43^. Here, we found that TNNT2 was more abundant (>twofold) than TNNI3 in all cavities and that their expression largely correlated across regions (Pearson correlation: 0.99). Interestingly, the cardiac isoform myosin-binding protein C3 (MYBPC3), which participates in stabilizing sarcomere structures, displayed a strikingly similar protein expression profile to cardiac troponins and was similarly abundant to TNNT2 (about 1.5-fold higher abundant) (Supplementary Fig. 8). Moreover, it can be detected by antibody- and MS-based approaches in human plasma after myocardial injury^44,45^, showing that it can be used as an useful additional parameter to monitor myocardial infarct.

Finally, while the RV and LV did not show any significantly altered protein expression, the protein myotilin, which stabilizes thin filaments during muscle contraction was much more abundant in LA compared to RA (>100-fold; Supplementary Fig. 9).

### Large vs. small arteries proteomes

Coronary arteries are relatively small as they supply the heart itself with blood; however, their malfunction is responsible for the high prevalence of coronary artery diseases (CADs), affecting more than 16 million individuals in the United States alone^46^. Overall, 1233 (13.8%) of quantified proteins were significantly more abundant in coronary arteries (RCA and LCA) and 631 (7.1%) in large arteries (Ao and PA) (Fig. 4b). Proteins involved in mitochondrial functions, collagen proteins, and integrins, such as COL15A1 and ITGB4 were highly enriched in the coronary vs. large arteries. Fibronectin (FN1) showed a 12-fold increase and has been previously associated with CADs^47^, although it would need to be investigated whether or not it is a specific marker for CAD patients. Arylsulfatase E (ARSE) is a constituent of artery walls where it regulates the composition of cartilage, and we found it to be >20-fold more abundant in coronary arteries.

Large arteries showed significant (*p* < 3 × 10^−42^) enrichment for cytoskeleton proteins and proteins involved in cell junction, consistent with the higher structural demands on them. For instance, fibulin 5 (FBLN5) was six- to tenfold higher expressed in PA and Ao than the other heart compartments (Supplementary Fig. 10). It is required to form the elastic lamina, has a protective role against vascular injury, and its downregulation has been associated with aortic aneurysm^48^. Interestingly, fibroblast growth factor 2 (FGF2) and ras-related protein (RAP1A), described as a key regulator of FGF2-induced angiogenesis^49^, were three- to sixfold more abundant in the large arteries. As we had not identified FGF2 in a deep plasma proteome previously^50^, it is unlikely to derive from blood remnants and may instead represent an ECM-bound form^51^. A total of 92 medium- to high- abundance proteins were exclusively quantified in the large arteries (Supplementary Data 4). This included the key focal adhesion protein SORBS1, COL26A1, and MYLK and MYH11, which are both involved in smooth muscle contraction, reflecting the higher proportion of SMCs in the large arteries’ wall compared to coronary arteries.

### The atrioventricular vs. semilunar valves proteome

The atrioventricular valves separating atria from ventricles (TV and MV) are morphologically quite different from the semilunar valves (AV and PV) preventing backflow of blood from aorta or pulmonary artery to ventricles. We found that only 497 (5.6%) proteins were significantly more abundant in the atrioventricular valves and 242 (2.7%) were more abundant in the semilunar valves (Fig. 4c). Valves are composed of highly organized ECM proteins and changes in its composition and their possible release are expected during valve deterioration, leading to dysfunction and failing heart valves^52–54^. Although the overall changes were limited, we found that among the ECM proteins, plectin isoform 3 and GFAP were highly expressed in the semilunar valves, whereas peripheral plasma membrane CASK, collagen enzyme P4HA1, integrin ITGA8, and neurofibromin (NF1) were significantly higher in atrioventricular valves.

### Cell-type-resolved proteome of the human heart

**Fig. 5:**
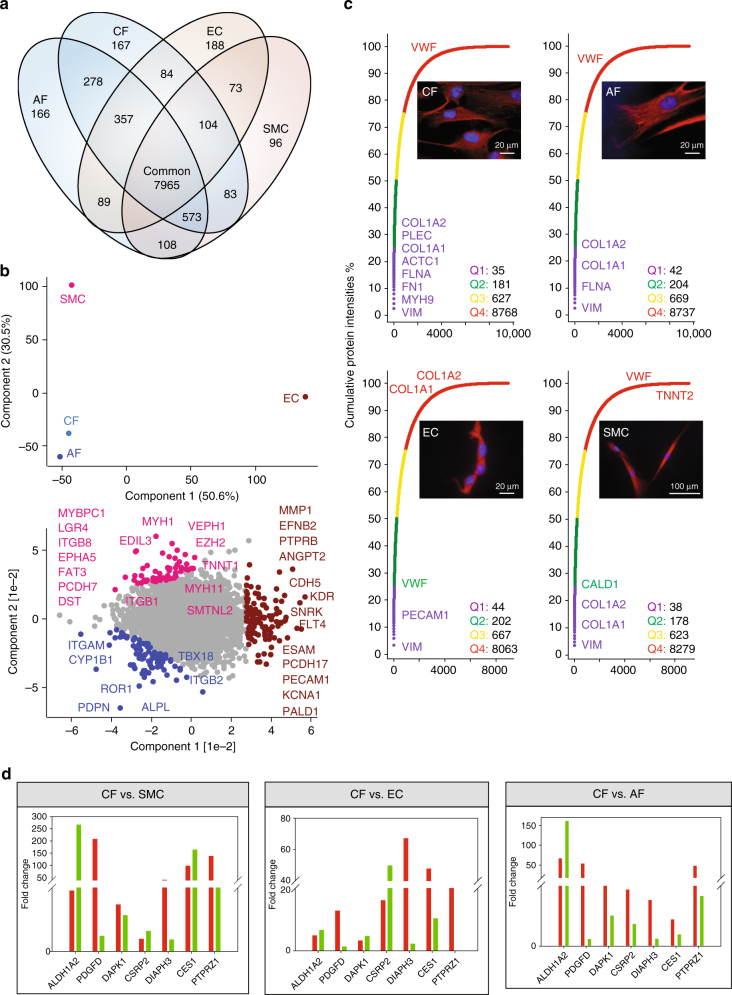
Comparative analysis of the cell-type proteomes. **a** Commonly and exclusively quantified proteins in three cardiac cell types and adipose fibroblasts. **b** PCA comparing the four cell types based on component 1 and 2, which accounted for 50.6 and 30.5% of the variability, respectively. **c** Cumulative protein abundances for each cell type and total number of proteins constituting the quantiles (Q1–Q4). The corresponding cell types are illustrated with immunofluorescence pictures at ×100 magnification. **d** RT-qPCR (green) and proteomic (red) fold-changes of the indicated genes in CF compared to all other cell types

Our region-resolved proteome achieved great depths, but as we used homogenized tissue, we do not have cell-type-specific information about the origin of the proteins. To address this, we isolated CFs, ECs, and SMCs from tissue samples harvested during cardiac surgery (Supplementary Table 2, “Methods” section). AFs were included to help in defining the CF-specific proteome. Cardiomyocytes were not investigated because of the impossibility to culture these cells from surgical biopsies. We achieved highly purified cell populations with values for CFs, AFs, ECs, and SMCs of 96%, 97%, 96%, and 92%, respectively (Supplementary Fig. 11a–d). Of a total of 11,236 different proteins, 7965 were identified in all four cell types, indicating that the majority of the cardiac cell proteome is expressed in its major cell types (Fig. 5a). We found high correlation (0.92) in protein expression between the fibroblast cell types (CF and AF), whereas SMC and EC were somewhat less related (0.81) and this is also reflected in the PCA (Supplementary Fig. 12 and Fig. 5b). On average, the 40 most abundant proteins accounted for 25% of the total protein mass in all four cell types (Fig. 5c). Consistent with the mesodermal origin of these cell types, vimentin (VIM) was the most abundant protein, accounting for 3% of the total protein mass. In conjunction with LARP6, VIM stabilizes type I collagen mRNAs, leading to upregulation of the collagens COL1A1 and COL1A2^55^. We found the collagens in the top quartile (Q1) of expression in CFs, AFs, and SMCs, whereas they were among the least abundant proteins (Q4) in ECs. Cell-type-enriched proteins—those with at least twofold higher expression in one of the cell types compared to all others—are listed in Supplementary Data 7.

### CF-enriched cell surface markers

Over the past decade, CFs have been shown to hold great promise as a potential target population for cardiac regenerative therapies^1,56,57^. Selection and targeting of CFs, however, remains challenging and currently relies on unspecific CF markers, including VIM, discoidin domain-containing receptor 2 (DDR2), periostin (POSTN), protein S100A4, ACTA2, platelet-derived growth factor receptors PDGFRα and β, T-box transcription factor TBX18, and the THY1 membrane glycoprotein^58^. Among these, only PDGFRB, S100A4, and ACTA2 showed at least twofold enrichment in CFs compared to ECs and SMCs, whereas all other currently employed CF markers were not enriched in CFs. Remarkably, compared to another fibroblast cell type (AFs), none of these markers were even twofold enriched. GATA4 and TBX20 have been reported as specific CF markers^59^. GATA4 was indeed only identified and quantified in CFs; however, TBX20 was fourfold more abundant in SMCs compared to CFs.

Globally, 609 (5.8%) proteins were specifically enriched in CFs compared to AFs, ECs, and SMCs. These encompassed 25 cell membrane receptors (Supplementary Data 8). The presence of the tyrosine kinase ROR1 in CFs has not been reported before but we found it to be 200-fold more abundant than in the other cardiac cell types. Activin receptor ACVR1 is required for normal heart development^60^ and it was also one of the most highest expressed proteins in CFs. Natriuretic peptide receptor NPR3 has a central role in vasodilatation, is known to be present in CFs,^61^ and our data showed sixfold higher expression in CFs. The drug target hepatocyte growth factor receptor MET, was also increased in CFs (fourfold higher than ECs and SMCs). BDKRB2, the receptor for bradykinin plays a pivotal role in the cardiovascular system by regulating blood pressure. Interestingly, we exclusively identified it in fibroblasts, with 11-fold higher expression in CFs compared to AFs. Likewise, protein levels of the cell membrane phosphatase PTPRZ1 were more than 40-fold higher in CFs compared to all other investigated cell types, a finding supported by qPCR. Interestingly, the direction of expression changes was concordant between the mRNA and protein levels. Importantly, however, for this and six other genes, the fold-changes indicated by qPCR were not predictive of the actual protein-level changes (Fig. 5d). Our data provide a catalog of CF-enriched marker candidates that hold promise for better definition and targeting of human CFs.

### EC and SMC proteome

Two further major cell types of the human heart include ECs, which form the inner lining of heart blood vessels and SMCs, the major constituents of the heart vasculature. ECs contain numerous storage granules filled with von Willebrand factor (VWF), which is involved in hemostasis. VWF was one of the most abundant proteins in ECs, whereas it was among the least abundant in CFs, AFs, and SMCs (Fig. 5c). The platelet endothelial cell adhesion molecule (PECAM1/CD31) was among the most abundant (Q1) proteins in ECs. Furthermore, EC- specific proteins ESAM (>22-fold) and ESM1 (exclusively) were overrepresented. Proteins involved in blood vessel morphogenesis such as the VEGF receptors FLT1, FLT4, KDR, as well as EPHB4 and its ligand Ephrin B2 showed 3- to 250-fold higher expressions compared to the other cell types. Comparing our data with single- cell transcriptomic data^62^ revealed that all genes identified as EC specific compared to CF (except CAV2, which only displayed a moderate increase) also showed at least several 10-fold upregulation in ECs at the proteomic level (Supplementary Data 8).SMCs contain the same muscle-contracting proteins as cardiac cells but do not have troponin. The low levels of cardiac TNNT2 detected (>100-fold less than in cavities), are likely due to the 8% impurity of isolated SMCs. In place of troponins, caldesmon (CALD1), which was among the highest abundant proteins in SMCs, blocks the myosin-head binding site on actin filaments^63^. Proteins segregating the SMC group (Fig. 5b, highlighted in pink) from EC, AF, and CF included typical smooth muscle proteins such as SMTNL2 and MYH11 of which we quantified three splice variants and two were exclusively identified in SMCs. Our analysis also opens up for the investigation of new epigenetic mechanisms, for instance, based on the very significant enrichment for EZH2-regulated proteins (*p* < 10^−34^).

### Clinical application of the heart map to atrial fibrillation

**Fig. 6:**
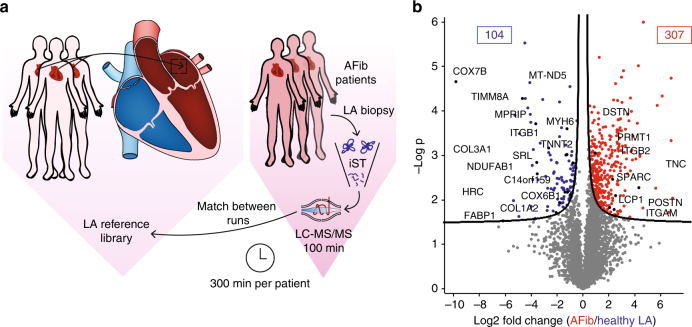
Clinical application of the healthy human heart atlas to atrial fibrillation. **a** Experimental workflow: LA tissues from three atrial fibrillation patients (AFib) were single-runs of technical triplicates. Data were matched against the healthy human LA library. **b** Volcano plot of the *p* values vs. the log2 protein abundance differences in AFib compared to healthy LA. Significantly up- and downregulated proteins are highlighted in red and blue, respectively (FDR 0.05)

Having generated a map of the healthy human heart, we next investigated if it could serve as a reference to pinpoint molecular differences between healthy and diseased tissue. To this end, we collected LA samples from three patients suffering from AFib (Supplementary Table 3), the most common heart arrhythmia and a major cause of mortality^64^. We applied a single-run method, in which an in-depth measurement of the proteomic system in question serves as a reference set of identified peptides for deep and high-throughput single-run measurements^65^ to assess the proteomic changes in AFib patients from minimal material and in a timely manner. We found that combining the iST sample preparation, our established healthy reference heart “library,” and single-run, triplicate measurements, any cardiac sample can be profiled in less than two days, of which only 300 min are MS-measuring time (Fig. 6a). In this way, we quantified an average of 3681 proteins for the healthy LAs and 4147 proteins in the AFib group, with excellent average Pearson correlations for technical and “biological replicates” of 0.97 and 0.93, respectively (Supplementary Fig. 13). In the AFib group compared to healthy samples, 104 proteins were significantly downregulated and 307 were upregulated (Fig. 6b and Supplementary Data 9). Proteins with increased expression in AFib are involved in ribonucleoprotein complexes and transcription. Some ECM proteins, such as DSTN, ITGB2, ITGAM, and FLNB, were significantly upregulated in the AFib group, whereas others, including COL1A2, COL3A1, and ITGB1 were downregulated. These results point to a reorganization of the ECM in AFib, explaining previous observations at the level of expressed proteins^66^. There is also evidence of significant contractile remodeling in AFib with several-fold lower expression of TNNT2, HRC, MYH6, SCN5A, and SRL, suggesting disruption of the cardiac tissue. Furthermore, MPRIP, a protein that has been previously associated with an increased number of stress fibers when downregulated^67^ showed 12-fold lower expression. The most significantly downregulated proteins in the AFib group (Fig. 6b and lower yellow box in Supplementary Fig. 14) were enriched for “mitochondrion” (*p* < 10^−100^). This included the most significantly downregulated protein (COX7B) and two other key mitochondrial proteins—IMMT and TIMM8A, all of which were >25-fold less abundant than in healthy tissue. Mitochondrial dysfunction has already been reported in AFib^68–71^; however, the broader molecular nature of their defects and whether they are different between patients are not fully understood. Interestingly, inspection of the hierarchical clustering plot within the AFib group revealed distinct and nonoverlapping clusters of up- and downregulated proteins for each of the three patients (orange and green clusters, Supplementary Fig. 14), whereas nondiseased biological controls showed similar expression to each other. Although the number of AFib patients is much too small to derive a general signature, our data clearly show that the mitochondrial defects reflected in the proteomes are very different between individuals. These proteomic patterns point to a potential molecular subclassification of AFib patients. These observations, however, will require a more thorough analysis including a larger patient cohort.

## Discussion

Creating anatomical and cellular maps increases our understanding of human biology and diseases^15,16,72^. Here, we used “loss-less” high-pH reversed-phase fractionation and high resolution, quantitative MS to generate a heart region, and cell-type-resolved human heart map. Starting from low microgram sample amounts, we quantified over 11,000 proteins, representing by far the deepest proteome of the healthy human heart, which is available in the online database MaxQB^25^. The ability to work with minimal starting material enables in-depth proteomics analyses from heart biopsies that can be obtained during surgery. Furthermore, our streamlined proteomics workflow enables the profiling of any cardiac sample in less than two days, a realistic time frame for future clinical application. At the anatomic level, we found that the 16 heart regions clustered into the expected three main areas (cavities, vessels, and valves). Binary comparison of subgroups, such as the atria and ventricles, provides crucial information to understand the basis for atrioventricular differences in healthy as well as diseased human hearts, a precondition to identify more specific and reliable biomarkers. To complement the region-resolved heat map, we also established a comprehensive proteomic map of three noncardiomyocyte cell types. This should be particularly useful in future studies to better define and target the CF population. CFs are activated into myofibroblasts after acute myocardial infarction, leading to increased ECM production and wound healing by scar formation within the infarction area. The direct reprogramming of resident CFs after myocardial infarction into induced cardiomyocytes or cardiac progenitor cells is currently a promising strategy for cardiac regeneration. Our quantitative proteomic data question the specificity of currently used CF markers while providing a promising panel of enriched cell surface markers for therapy. There is a lack of reliable biomarkers for aortic aneurysms (enlargement of the Ao) or more importantly aortic dissection (tear in the wall of the Ao). Currently, both diagnoses rely on clinical examinations and laborious imaging techniques. Our deep quantitative proteome of large arteries, in particular the Ao, could help in establishing a healthy baseline in future studies aiming to define protein expression indicative of these conditions. Likewise, our human cardiac valve proteome can be used as a background for future studies aiming to uncover biomarkers indicative of cardiac valve deterioration. Finally, we show that patients suffering from AFib present both common and distinct proteome profiles, potentially pointing to individual-specific disease manifestation. While our investigation is only a first step, it opens up as a yet-unexplored molecular classification at the level of expressed proteins. Further directions in human heart proteomics could include region- and cell-type-specific mapping at higher resolution, investigation of PTMs, and the combination of proteomics with detailed mechanistic investigations of disease etiology.

## Methods

### Tissue preparation

In total, 16 healthy heart regions from three adult male individuals (Supplementary Table 1) were collected less than 72 h postmortem during an autopsy after a court order. The hearts of the subjects did not present any relevant injury or signs of cardiac malfunction and were therefore defined as healthy. The 16 heart regions included four main vessels (aorta (Ao), pulmonary artery (PA), vena cava inferior (IVC), and pulmonary vein (PVe)), four heart cavities (right atrium (RA), left atrium (LA), right ventricle (RV), and left ventricle (LV)), four heart valves (tricuspid valve (TV), pulmonary valve (PV), aortic valve (AV), and mitral valve (MV)), the ventricular septum (SepV), the atrial septum (SepA), the left coronary artery (LCA), and the right coronary artery (RCA) were explanted by an official medicolegal expert. Samples were stored at −80 °C after collection. The investigation was approved by the local ethical committee of the Medical Faculty of the Technical University of Munich (project no. 247/16s). The ethical committee explicitly approved the use of human samples in the context of trauma.

### Cell isolation

Atrial samples from patients undergoing cardiovascular surgery were cut into 1–2-mm^2^ fragments and digested with 2 mg per ml of collagenase type II (Life Technologies, Cat. No. 17101-015, Carlsbad, CA) in PBS (1 h, 37 °C). After filtration (70-µm cell strainer (Greiner Bio-One, Cat. No. 542070, Frickenhausen, Germany) and red cell lysis (Red Blood Cell Lysis Solution, Miltenyi Biotec, Cat. No. 130-094-183, Bergisch Gladbach, Germany), the remaining cells were resuspended in 1 ml of auto-running MACS buffer (Miltenyi Biotec, Cat. No. 130-090-221). After preseparation (30-µm filter, Miltenyi Biotec, Cat. No. 130-041-407), CD31-positive endothelial cells (ECs) were isolated using the CD31 MicroBead Kit (Miltenyi Biotec, Cat. No. 130-091-935) and the human FcR-Blocking Regent (Miltenyi Biotec, Cat.No. 130-059-901) in the MACS system (Miltenyi Biotec) according to the manufacturer’s instruction. Isolated ECs were cultured in Endothelial Cell Growth Medium 2 (PromoCell, Cat. No. C-22011, Heidelberg, Germany) until confluence. Adipose fibroblasts (AF) were isolated from subcutaneous fat tissue and cardiac fibroblasts (CF) were isolated from atrial samples. Tissues were cut and digested using collagenase solution type II (2.5 h, 37 °C), and resuspended in DMEM high glucose (Biochrom-Millipore, Cat. No. FG 0435; Berlin, Germany) containing 10% fetal calf serum (FCS, Fisher Scientific, Schwerte, Germany), penicillin (100 U per ml), streptomycin (100 µg per ml), both from PanReacAppliChem (AppliChem, Darmstadt, Germany), and sodium-pyruvate (1 mM, Gibco, Karlsbaden, CA) and grown to confluence. Smooth muscle cells (SMCs) were isolated from the arteria mammaria interna (ITA). Vessels were cut longitudinally. With a scalpel, the tissue was cut into square pieces. The pieces were put on BD Primaria^TM^ 6-well plates (Greiner Bio-One, Frickenhausen, Germany) and dried (2–3 h, 37 °C, 5% CO_2_). Subsequently, SMC growth medium 2 (PromoCell GmbH, Heidelberg, Germany) was added. When the first SMCs migrated from the tissue, the pieces were removed and cells were grown to confluence. All biopsies from patients undergoing cardiovascular surgery were transferred within 10 min from the operation room in PBS. To passage the ECs, SMCs, and fibroblasts, cells were incubated with trypsin (Life Technologies) diluted 1 in 4 in PBS (5 min, 37 °C, 5% CO_2_). Trypsin solution was neutralized using 2 times of media. ECs and SMCs were frozen in liquid nitrogen in 10% DMSO (Roth, Karlsruhe, Germany), 40% FCS, and 50% cell-type-specific medium. CFs and AFs were frozen in liquid nitrogen using 10% DMSO and 10% FCS in fibroblast-specific medium as described above. The generation of cell cultures from patient biopsies was approved by the local ethical committee of the Medical Faculty of the Technical University of Munich (project no. 1588/06 (amendment) and 2919/10).

### Flow cytometry-based quantification of the cell types

Frozen SMCs isolated from the ITA (passage p2) and cultured as described above were thawed and used for flow cytometry analysis. SMCs were fixed in 1% formaldehyde (20 min) and blocked in PBS containing 10% FCS (v/v) and 0.05% sodium azide (w/v). The first antibody anti-alpha smooth muscle actin (ab5694, Abcam, UK) was added at a ratio of 1:20 in wash/permeabilization buffer (PBS containing 5% FCS (v/v), saponin 0.5% (w/v), and sodium azide (0.05%, w/v)) for 30 min at 4 °C. The secondary antibody goat-anti-rabbit Alexa Fluor 488^®^ (ab150077, Abcam, UK) was added at a ratio of 1:2000 in buffer containing saponin (30 min, ice, dark). ECs from atrial biopsies were isolated and cultured as described above. After preparation of a single-cell suspension, one sample of the total biopsy and the positive and negative fraction obtained after MACS were analyzed. All cell fractions were resuspended in ice-cold PBS/0.5% BSA/2 mM EDTA (FACS buffer) containing 5% anti-human CD31 PE-Cy7 (25-0319, eBioscience, Frankfurt, Germany) and incubated for 30 min in the dark. CFs and AFs were isolated as described before. Cells were cultured on gelatin-coated plates and analyzed at passage 1 or 2. Cells were detached using 0.25% trypsin (Life Technologies) diluted at a ratio of 1:4 in PBS. Monoclonal anti-human CD90 PE-Cy5 (eBioscience, 15-0909), anti-human CD105 APC (eBioscience, 17-1057), and anti-human CD45 FITC (eBioscience, 11-9459) antibodies were resuspended at a ratio of 1:20 in ice-cold FACS buffer and incubated for 30 min on ice in the dark. After staining, all cells undergoing flow cytometry analyses were resuspended in ice-cold PBS/0.5% BSA/4 mM EDTA and kept in the dark on ice until flow cytometric analysis was performed with a BD LSRFortessa (BD, San Jose, CA). Negative controls were unstained or stained with the secondary antibody alone. Cytometry data were analyzed with the FlowJo software version 7.6.5 (flowjo@treestar.com).

### Immunocytochemistry

SMCs were fixed (4% PFA, 20 min) and permeabilized in PBS-T (0.1% Triton-X-100 in PBS, 10 min). Unspecific binding was blocked with 5% normal goat serum (Abcam, ab7841, Cambridge, UK) in PBST for 30 min. Polyclonal rabbit anti-alpha smooth muscle actin (Abcam, ab5694) was diluted at a ratio of 1:20 in PBS-T and SMCs were incubated with the first antibody overnight at 4 °C. The secondary antibody goat-anti- rabbit IgG (H&L) Alexa Fluor 555^®^ (Abcam, ab150078) was diluted at a ratio of 1:200 in PBS-T. Cells were incubated in the dark for 60 min. ECs isolated from LA and the positive and the negative fraction after MACS sort were stained for CD31. Cells were isolated and processed using the MACS system as described above and plated on cover slides until confluence. Cells were fixed using 4% PFA (10 min) and blocked (5% goat serum in PBS). Polyclonal rabbit anti-CD31 (Abcam, ab28364) was diluted at a ratio of 1:25 and incubated overnight at 4 °C. Secondary antibody goat-anti-rabbit IgG (H&L) Alexa Fluor 555^®^ (Abcam, ab150078) was diluted at a ratio of 1:500 and incubated (60 min, dark). Both antibodies were diluted in PBS. CFs and AFs were fixed using 4% PFA/sucrose in PBS (15 min). For VIM staining, cells were permeabilized (0.25% Triton-X-100 in PBS) and unspecific binding sites were blocked (5% goat serum in PBS-T, 1 h). Polyclonal rabbit anti-VIM (Abcam, ab45939) was used as cytoskeleton marker at 1 µg per ml of final concentration in PBS-T. For DDR2, staining cells were washed with PBS after fixation and blocked with 5% goat-serum in PBS. Polyclonal rabbit anti-DDR2 (LSBio, LS-C99151, Seattle, WA) was diluted at a ratio of 1:20 in PBS. Both antibodies were incubated for 1 h. Secondary antibody goat-anti-rabbit IgG (H&L) Alexa Fluor 555^®^ (Abcam, ab150078) was diluted at a ratio of 1:500 in either PBS-T or PBS and incubated for 1 h in the dark. For immunocytochemistry, SMCs, ECs, CFs, and AFs were grown to approximately 80% confluence on 4-well chamber cover slides (Millizell EZ slides, Millipore, Darmstadt, Germany). All incubations were performed at room temperature, except overnight incubations. After the last wash, slides were air-dried, mounted in Abcam-mounting medium containing DAPI (Abcam, ab104139), sealed with coverslips, and evaluated under a fluorescent microscope (Axiovert 200 M, Zeiss, D-73447 Oberkochen).

### RT-qPCR analysis

Expression of CF-specific proteins was confirmed on transcriptional level by RT-qPCR analysis. ECs, SMCs, CFs, and AFs (p0-4) were lysed with RNA lysis buffer (Peqlab, Erlangen, Germany). Total RNA was purified using the peqGOLD total RNA kit (Peqlab) and reverse-transcribed into cDNA with M-MLV reverse transcriptase (Invitrogen, Darmstadt, Germany) according to the manufacturer’s recommendation. Expression of ALDH1A2, PDFD, DAPK1, CSRP2, DIAPH3, CES1, and PTPRZ1 was evaluated on a QuantStudio3 (Applied Biosystems, Foster City, CA) using Power SYBR Green Master Mix (Applied Biosystems) and the following conditions: activation of *Taq* DNA polymerase (15 min at 95 °C) followed by 40 cycles with 15 s at 95 °C, 60 s at 60 °C. The sequences of the used primers are noted in Supplementary Table 4. Quantification was performed using the relative expression software tool REST©. Data were normalized to β-actin.

### Sample preparation for MS analysis

All 16 heart regions dissected from three trauma victims were washed three times with cold PBS before being crushed in liquid nitrogen using a mortar and pestle. Powdered samples were then resupended in 500 µl of SDC reduction and alkylation buffer and boiled for 10 min to denature proteins^19^. Samples were further mixed (six times for 30 s and cooled on ice in-between) using a FastPrep^®^-24 Instrument (MP Biomedicals). Protein concentration was measured using the Tryptophan assay and 300 µg were further processed for overnight digestion by adding Lys-C and trypsin in a 1:50 ratio (µg of enzyme to µg of protein) at 37 °C and 1700 rpm. On the following day, samples were sonicated using a bioruptor (15 cycles of 30 s) and further digested for 3 h with Lys-C and trypsin (1:100 ratio). Peptides were acidified to a final concentration of 0.1% trifluoroacetic acid (TFA) for SDB-RPS binding and 40 µg of peptides were loaded on four 14-gauge Stage-Tip plugs. Peptides were washed first with isopropanol/1% TFA (200 µl) and then 0.2% TFA (200 µl) using an in-house-made Stage-Tip centrifuge at 2000×*g*. Peptides were eluted with 60 µl of elution buffer (80% acetonitrile/1% ammonia) into auto sampler vials and dried at 60 °C using a SpeedVac centrifuge (Eppendorf, Concentrator plus). Peptides were resuspended in 2% acetonitrile/0.1% TFA and sonicated (Branson Ultrasonics, Ultrasonics Cleaner Model 2510) before peptide concentration estimation using the Nanodrop. About 30 µg of peptides of each sample were further fractionated into 54 fractions and concatenated into 8 fractions by high-pH reversed-phase fractionation using the recently described “loss-less” nano-fractionator^20^. CFs, AFs, ECs, and SMCs were processed similarly to the heart tissue samples without liquid nitrogen crushing and FastPrep^®^-24 Instrument.

### Liquid chromatography–MS analysis

Nanoflow LC–MS/MS analysis of tryptic peptides was conducted on a quadrupole Orbitrap mass spectrometer^73^ (Q Exactive HF, Thermo Fisher Scientific, Rockford, IL, USA) coupled to an EASYnLC 1200 ultra-high-pressure system (Thermo Fisher Scientific) via a nano-electrospray ion source. About 1 µg of peptides were loaded on a 40-cm HPLC-column (75-μm inner diameter; in-house packed using ReproSil-Pur C18-AQ 1.9-µm silica beads; Dr Maisch GmbH, Germany). Peptides were separated using a linear gradient from 2 to 20% B in 55 min and stepped up to 40% in 40 min followed by a 5 min wash at 98% B at 350 nl per min where solvent A was 0.1% formic acid and 5% DMSO in water and solvent B was 80% acetonitrile, 5% DMSO, and 0.1% formic acid in water. The total duration of the run was 100 min. Column temperature was kept at 60 °C by a peltier element-containing, in-house-developed oven. The mass spectrometer was operated in “top-15” data-dependent mode, collecting MS spectra in the Orbitrap mass analyzer (60,000 resolution, 300–1650 *m/z* range) with an automatic gain control (AGC) target of 3E6 and a maximum ion injection time of 15 ms. The most intense ions from the full scan were isolated with an isolation width of 1.5 *m/z*. Following higher-energy collisional dissociation (HCD) with a normalized collision energy (NCE) of 27%, MS/MS spectra were collected in the Orbitrap (15,000 resolution) with an AGC target of 5E4 and a maximum ion injection time of 25 ms. Precursor dynamic exclusion was enabled with a duration of 30 s. For clinical AFib samples, a “top-5” data-dependent acquisition method as described above was modified to increase the dynamic range on the MS1 level by including three segmented MS scans (12 segments each; total AGC target 1E6), covering a *m/z* range of 400–1200. MS1 resolution was set to 120,000 at *m/z* 200 throughout.

### MS data analysis

Tandem mass spectra were searched against the 2015 Uniprot human databases (UP000005640_9606 and UP000005640_9606_additional) using MaxQuant^21^ version 1.5.5.6 with a 1% FDR at the peptide and protein level, peptides with a minimum length of seven amino acids with carbamidomethylation as a fixed modification, and N-terminal acetylation and methionine oxidations as variable modifications. Enzyme specificity was set as C-terminal to arginine and lysine using trypsin as protease and a maximum of two missed cleavages were allowed in the database search. The maximum mass tolerance for precursor and fragment ions was 4.5 ppm and 20 ppm, respectively. If applicable, peptide identifications by MS/MS were transferred between runs to minimize missing values for quantification with a 0.7-min window after retention time alignment. Label-free quantification was performed with the MaxLFQ algorithm using a minimum ratio count of 1. For clinical AFib samples, the identification transfer was restricted to the healthy LA library only, and we set a minimum ratio count of 2 for label-free quantification.

### Statistical analysis

Statistical and bioinformatics analysis was performed with the Perseus software^32^ (version 1.5.5.0), Microsoft Excel, and R statistical software. Proteins that were identified in the decoy reverse database or only by site modification were not considered for data analysis. We also excluded potential contaminants. Data were further filtered to make sure that identified proteins showed expression in all biological triplicates of at least one heart region and missing values were imputed on the basis of normal distribution (down shift = 1.8, width = 0.15). PCA analysis of the heart region and cell types relied on singular value decomposition and the original feature (protein) space was orthogonally transformed into a set of linearly uncorrelated variables (principal components). These account for distinct types of variability in the data. For hierarchical clustering, LFQ intensities were first z-scored and clustered using Euclidean as a distance measure for column and row clustering. Gene set enrichment analysis (GSEA) was performed using gene set collections from the MSigDB^26^. Mean log2 ratios of biological triplicates and the corresponding *p* values were visualized with volcano plots. We used *t*-test for binary comparisons and SAM with *s*_0_ = 0.1 and FDR < 0.05 for the assessment of *t*-test results in volcano plots^32^.

### Copy number calculation and subcellular heart proteome model

Conversion of LFQ intensities to copy number estimations was achieved using the proteomic ruler^27^. The proteomic ruler plug-in v.0.1.6 was downloaded from the Perseus plug-in store, for use with Perseus version 1.5.5.0. Protein intensities were filtered for three valid values in at least one heart region. Proteins belonging to the GO term “blood microparticle” were removed from the analysis (see Supplementary Data 3 for a full list of removed proteins). Protein groups (proteins that can be distinguished based on the available peptide information) were annotated with amino acid sequence and tryptic peptide information for the leading protein ID, using the .FASTA file used for processing in MaxQuant. Copy numbers per diploid nucleus were estimated using the following settings: averaging mode—“All columns separately,” molecular masses—“average molecular mass,” detectability correction—“Number of theoretical peptides,” scaling mode—“Histone proteomic ruler,” ploidy “2,” and total cellular protein concentration—“200 g per l.” To build a subcellular model of the heart atlas proteome, subcellular localization predictions from spatial proteomics data^28,29^ were matched to the protein groups using the leading canonical protein ID. The median copy number of the three replicates was multiplied by the protein molecular weight to calculate protein mass. The mass of each protein was attributed to the nucleus, cytosol, or a specific organelle according to its distribution in HeLa cells. Since many highly abundant heart-specific proteins were not present in this spatial proteomics database, the top 100 proteins in each heart region were completed for subcellular localization using annotation from UniProt. These manually annotated protein masses were assigned entirely to the respective organelle. This led to a median of 94% of total protein mass being assigned to a specific location.

### Data availability

All MS proteomics data have been deposited on ProteomeXchange via the PRIDE database with the data set identifier PXD006675 and can also be accessed in a user-friendly format at maxqb.biochem.mpg.de. All other data supporting the findings of this study are available within this article and in the supplementary material or from the corresponding authors on reasonable request.

## Electronic supplementary material

Supplementary Information

Description of Additional Supplementary Files

Supplementary Data 1

Supplementary Data 2

Supplementary Data 3

Supplementary Data 4

Supplementary Data 5

Supplementary Data 6

Supplementary Data 7

Supplementary Data 8

Supplementary Data 9
