# Supplementary material for: Region and cell-type resolved quantitative proteomic map of the human heart
Source: Nat Commun. 2017 Nov 13;8:1469. doi: 10.1038/s41467-017-01747-2 (PMC5684139; doi:10.1038/s41467-017-01747-2)
Supplement: Supplementary file 1 — Supplementary Information [file 41467_2017_1747_MOESM1_ESM.pdf]

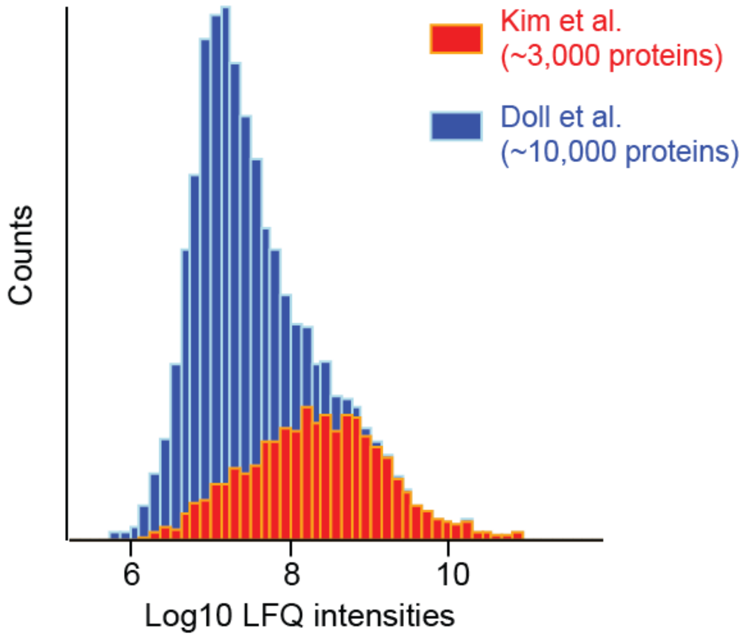

Supplementary Figure 1 Heart proteomic data comparison between the 'human draft proteome' and our data. The histogram shows the total proteins identified in our study (highlighted in blue) compared to the proteins identified in the Kim et al paper (highlighted in orange).

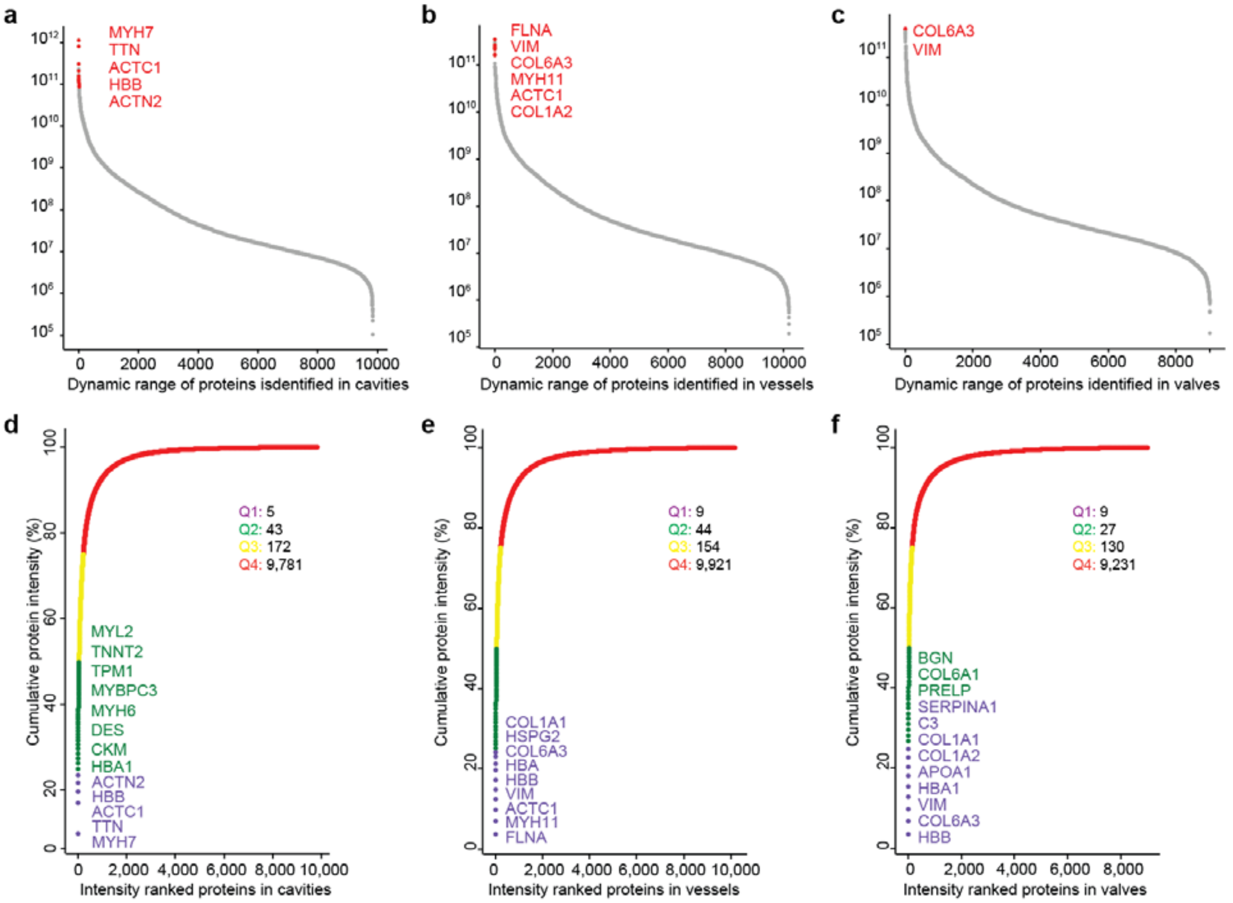

Supplementary Figure 2 Cumulative protein abundance and dynamic range in each heart area. Cumulative protein abundance in cavities (**a**), vessels (**b**), and valves (**c**). Dynamic range of proteins identified in cavities (**d**), vessels (**e**), and valves (**f**).

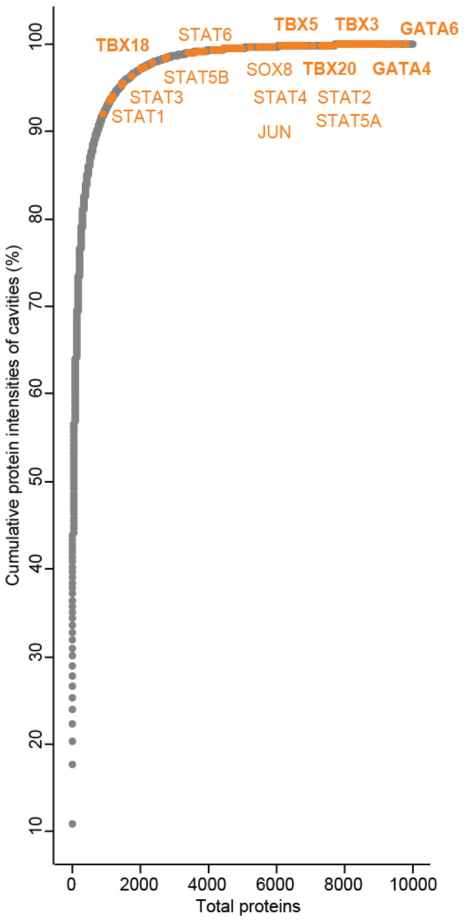

Supplementary Figure 3 Detection of low abundant transcription factors. Cumulative protein abundance in cavities shows the detection and quantification of low abundant transcription factors (highlighted in orange) in the last quantile.

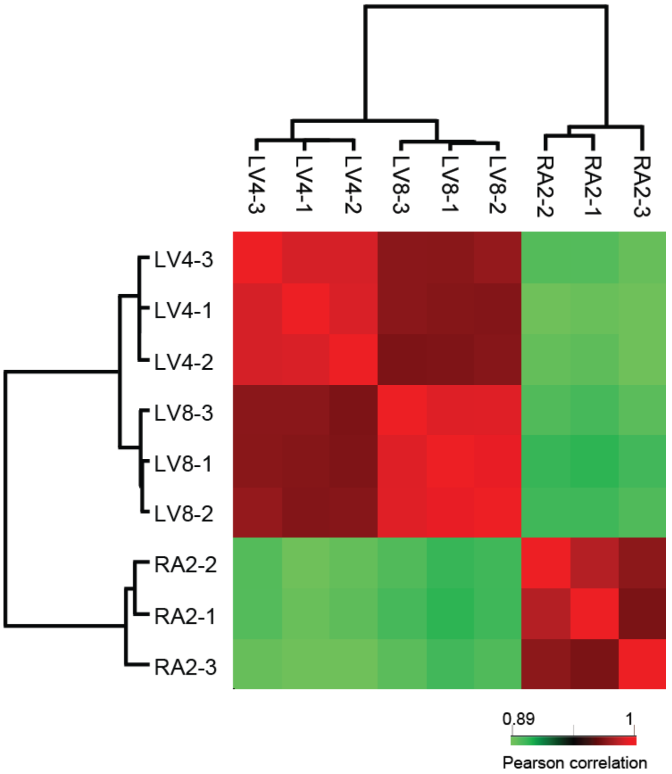

Supplementary Figure 4 Technical reproducibility. High and low Pearson correlations between each heart region are represented in red and green, respectively.

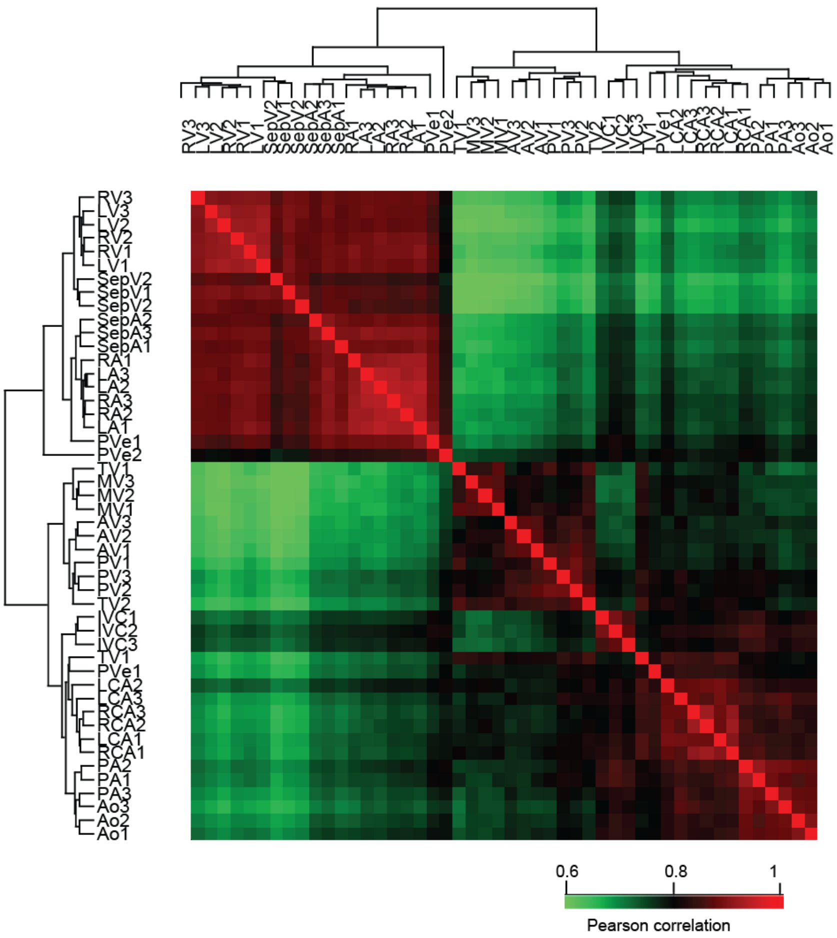

Supplementary Figure 5 Reproducibility between the different donors. High and low Pearson correlations are denoted in red and green, respectively.

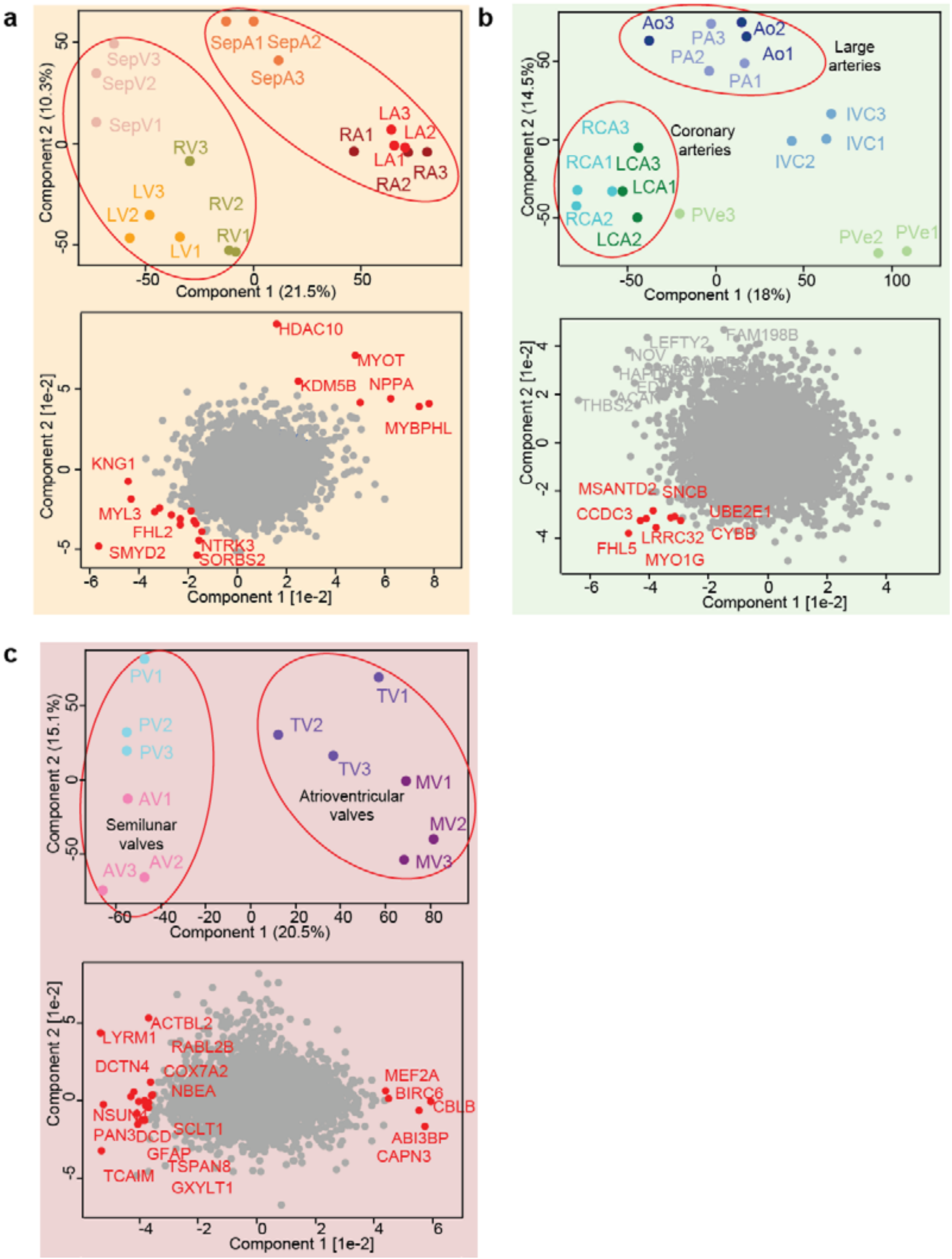

Supplementary Figure 6 PCA of the cavities, vessels, and valves based on their proteomic expression profiles. (**a**) The proteome of the cavities segregated into the ventricular (RV, LV, SepV) and atrial (RA, LA, SepA) part based on component 1 and 2, which accounted for 21.5% and 10.3% of the variability, respectively. (**b**) Vessels segregated into coronary arteries (RCA, LCA) and outgoing vessels (Ao, PA) based on component 1 and 2, which accounted for 18% and 14.5% of the variability, respectively. (**c**) Valves clustered into ventricular (MV, TV) and semilunar valves (AV, PV) based on component 1 and 2, which accounted for 20.5% and 15.1% of the variability.

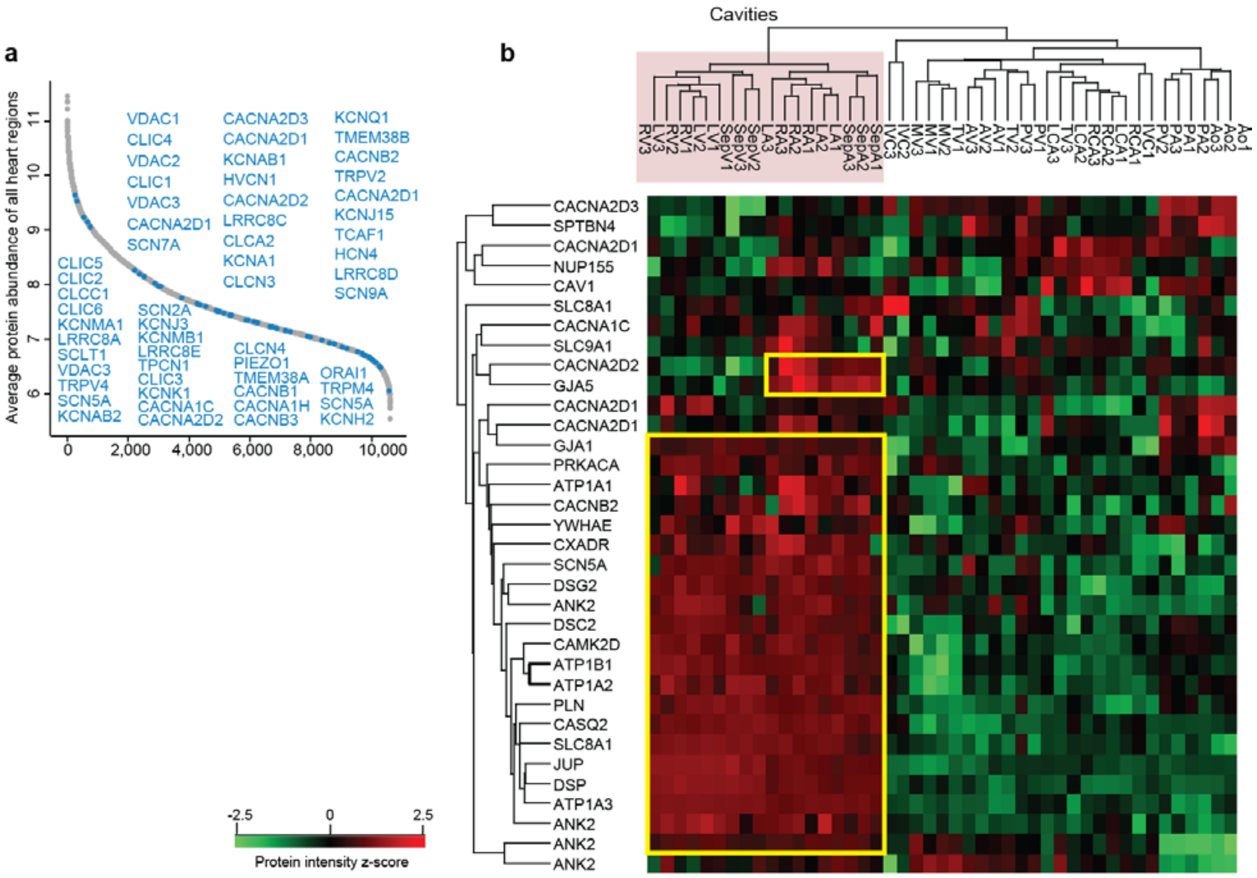

Supplementary Figure 7 Cardiac ion channels. (**a**) Proteins involved in the conduction of an electrical impulse across the heart (highlighted in blue). (**b**) Heat map of z-scored protein abundance (LFQ intensities) of the differentially expressed proteins (ANOVA, FDR < 0.05).

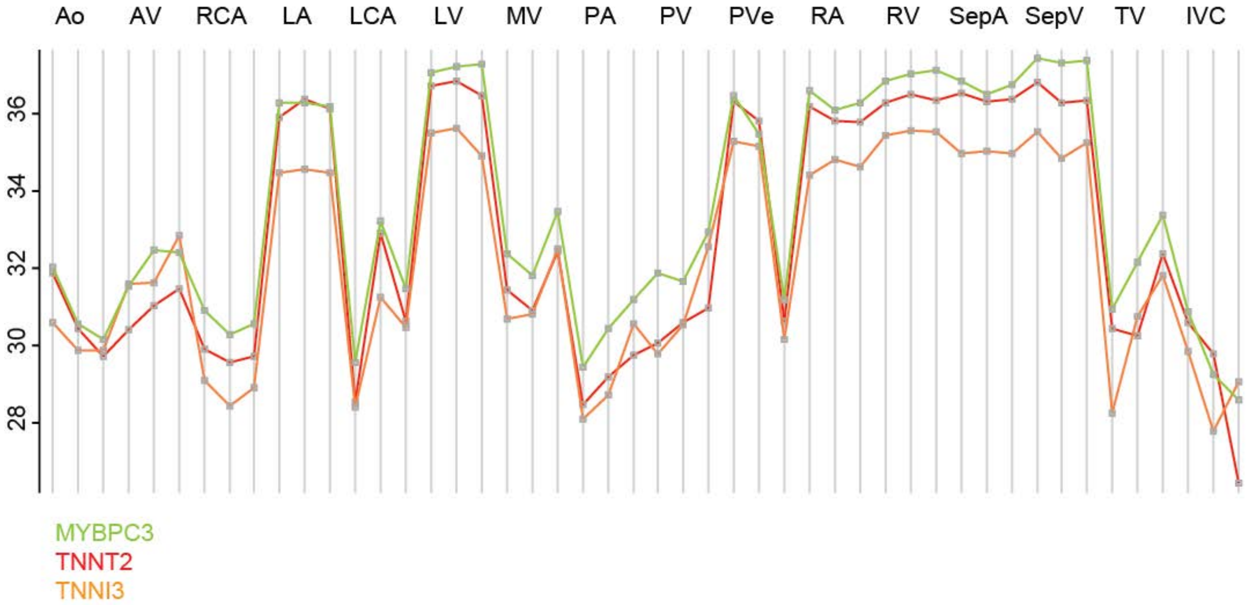

Supplementary Figure 8 MYBPC3 expression across all cardiac samples. Profile plot of the expression of MYPC3 (green),TNNT2 (red), and TNNI3 (orange) across all cardiac samples.

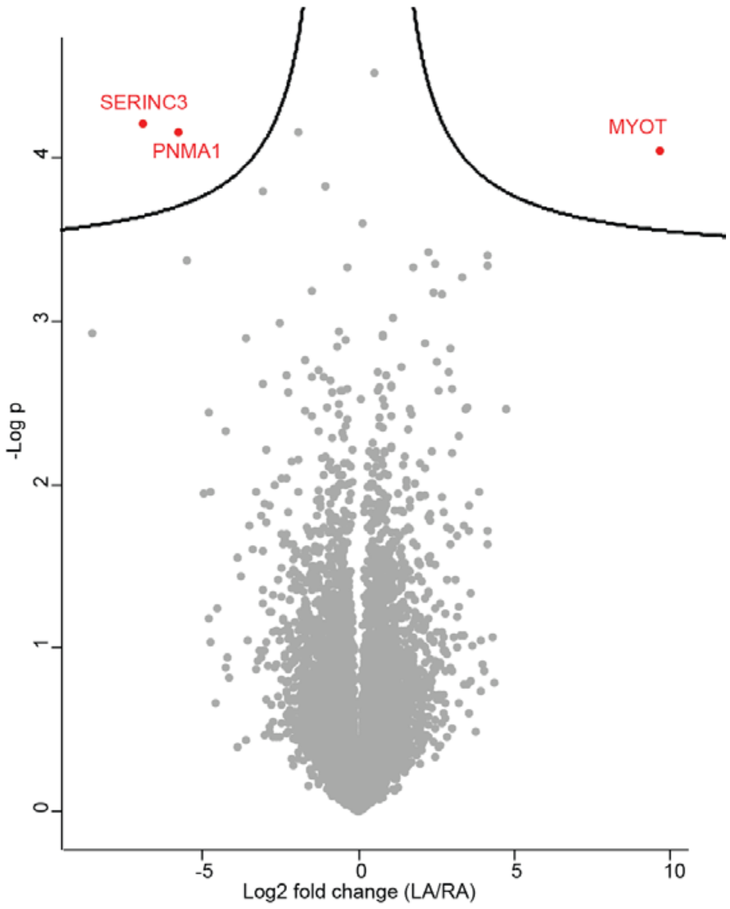

Supplementary Figure 9 Proteins differentially expressed in the right and left atrium. Volcano plot of the p-values versus the log2 protein abundance differences between the RA and LA.

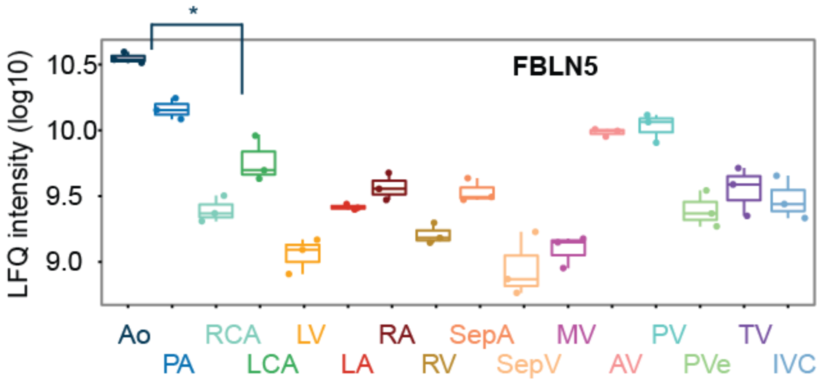

Supplementary Figure 10 Significant upregulation of FBLN5 in the aorta. Multiple t-tests show the significant upregulation (marked with an asterisks) of FBLN5 in the Ao (highlighted in dark blue) compared to all other heart compartments. Boxplots represent the three biological replicate variance. LFQ intensities (log10) are represented on the y-axis, while the different heart compartments are listed on the x-axis.

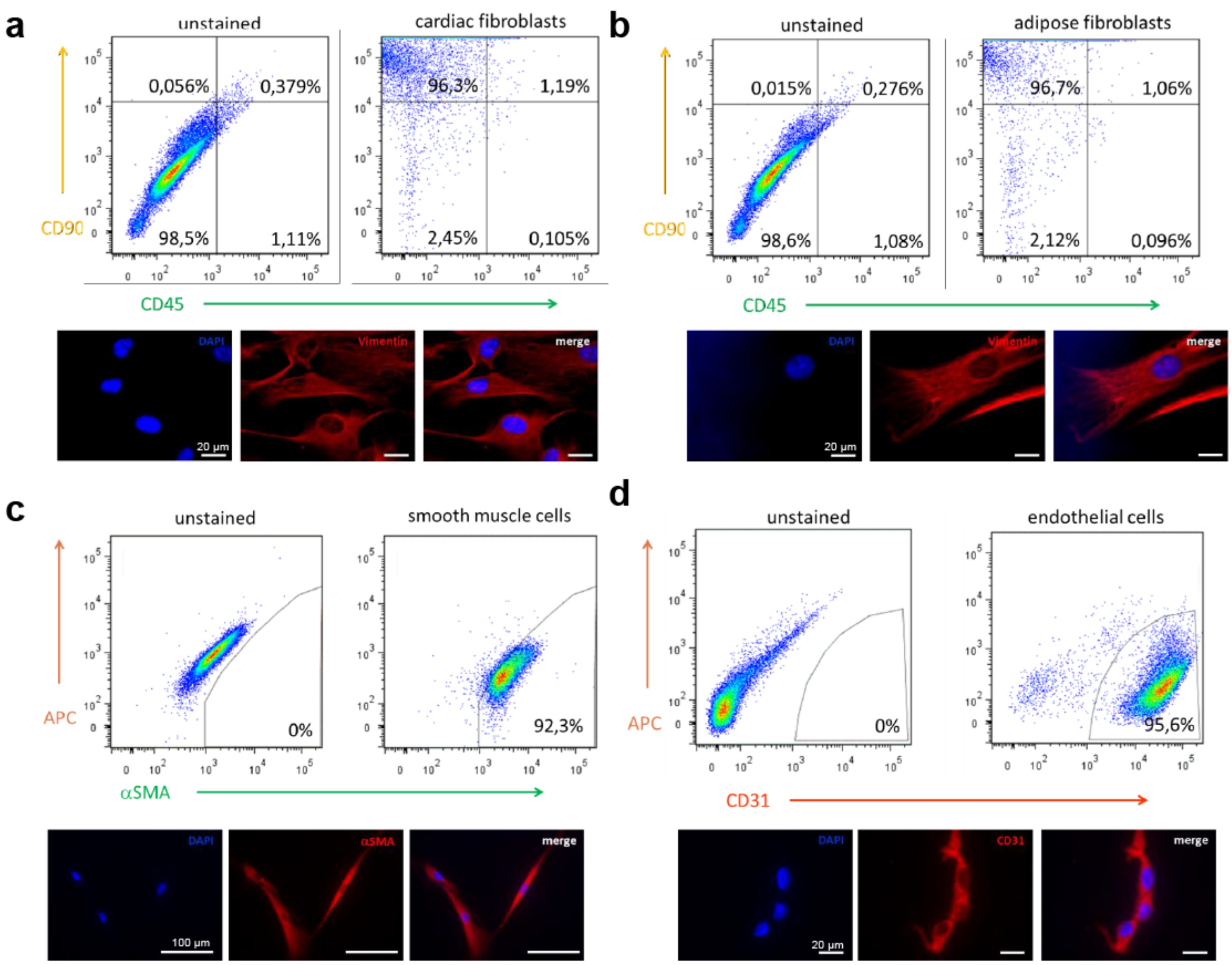

Supplementary Figure 11 Isolation and purity of CF, AF, SMCs, and ECs. FACS-based isolation of CFs (**a**), AFs (**b**), SMCs (**c**), and ECs (**d**). The FACS sorting results are represented for each cell type in the upper panel and corresponding microscopic images in the lower panel, where the scale bar represents 20 µm, except for SMCs (100 µm).

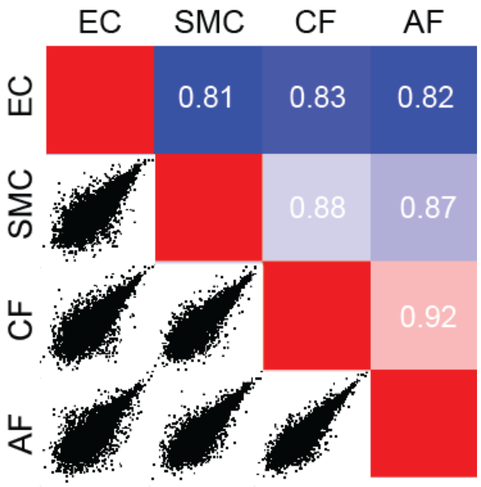

Supplementary Figure 12 Correlations between the cell lines. Pearson correlations between CF, AF, SMC, and EC.

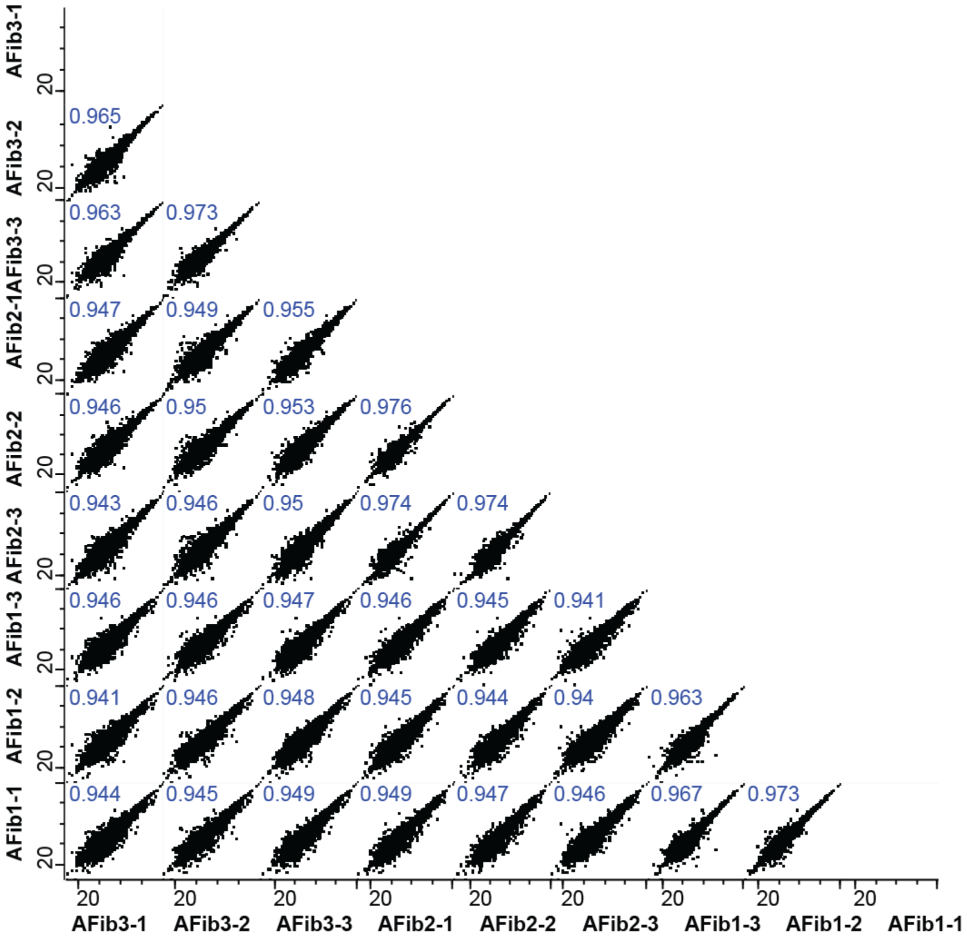

Supplementary Figure 13 Technical reproducibility of clinical measurements. Pearson correlations between technical triplicates of AFib patients (-1, -2, and -3).

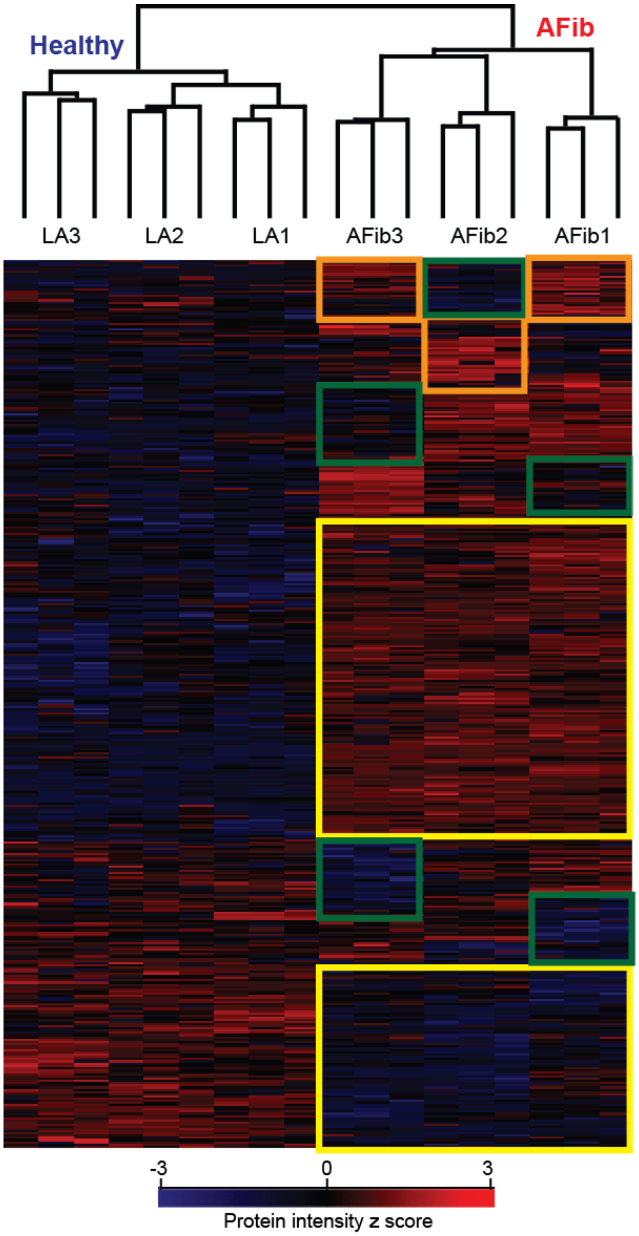

Supplementary Figure 14 Hierarchical clustering of the AFib vs. control group. Heat map of z-scored protein intensities (LFQ values) of the differentially expressed proteins in three AFib patients (AFib1, AFib2, AFib3) compared to three healthy human LAs (LA1, LA2, LA3) (ANOVA, FDR < 0.05). The upper yellow cluster highlights proteins commonly upregulated in AFib compared to normal and lower yellow cluster, those downregulated compared to normal. Orange and green boxes enclose proteins up- and down-regulated proteins of each of the patients compared to the other two. The color key denotes normalized protein expression values.

Subject	sex	age	body height [cm]	body mass [kg]	cause of death	Heart morphology
1	male	22	184	88	Suicide, intoxication	*NAD
2	male	21	178	84	Multiple trauma	*NAD
3	male	47	188	86	Multiple trauma	*NAD

***NAD no abnormality detected**

Supplementary Table 1 Characteristics of the donors

Patients	Sex	Age	Diagnosis
**KaBi-DHM 1**	male	66	coronary heart disease
**KaBi-DHM 2**	female	66	mitral regurgitation
**KaBi-DHM 3**	male	77	coronary heart disease
**KaBi-DHM 4**	male	68	coronary heart disease
**KaBi-DHM 5**	female	75	mitral regurgitation
**KaBi-DHM 6**	female	61	mitral regurgitation
**KaBi-DHM 7**	male	68	mitral regurgitation
**KaBi-DHM 8**	male	64	mitral regurgitation
**KaBi-DHM 9**	male	83	coronary heart disease, aortic regurgitation
**KaBi-DHM 10**	male	76	coronary heart disease

Cardiovascular biobank of the German Heart Centre (KaBi-DHM)

Supplementary Table 2 Characteristics of cell donors

				sides diagnoses												
patient	Sex	Age	Type atrial fibrillation (type AF)	arterial hypertension (AH)	diabetes mellitus type 2 (DM type 2)	hyperlipidemia (HL)	renal failure (RF)	coronary heart disease (CHD)	body mass index (BMI)	systolic pulmonary arterial pressure (syst. PAP)	size of left atrium	left ventricle function (LV function) ejection fraction	Interventricular Septal Thickness at Diastole (IVSd)	mitral valve annulus	Left ventricular end-diastolic diameter (LVED)	Tricuspid Annular Plane Systolic Excursion (TAPSE)
	[male/ female]	[years]								[mm Hg]	[mm]	[%]	[mm]	[mm]	[mm]	[mm]
**A3689 (1)**	female	76	persistent	no	no	yes	no	no	22	45,0	50	55	13	39	49	13
**A2719 (2)**	male	78	permanent	no	no	yes	no	no	21	32,0	53	47	12	44	53	16
**A2725 (3)**	female	74	persistent	no	no	no	no	no	27	41,0	54	45	22	38	49	24

Supplementary Table 3 Characteristics of the atrial fibrillation patients

**Target**		**Sequence**
β-actin	forward<br>reverse	CCA ACC GCG AGA AGA TGA<br>CCA GAG GCG TAC AGG GAT AG
ALDH1A2	forward<br>reverse	ATC AAC AAG GCC CTC ACA GT<br>TCT GGG CAT TTA AGG CAT TG
PDGFD	forward<br>reverse	TTC AAG ATT TAT TAT TCT TTG CTG GA<br>CCC CTG AAA TAG AGC TTG TGA
DAPK1	forward<br>reverse	CCC TTG TCC CAG TTG AAG AA<br>CCG GTC GAG GAA CAT TCA
CSRP2	forward<br>reverse	CTG ACT GAA AAA GAA GGT GAA ATC T<br>TTA CTG GGC ATG AAC AAG AGC
DIAPH3	forward<br>reverse	CTC CGG CAC AAT TCA GTT C<br>GAT GTG AAG CCT GAA ATC CAA
CES1	forward<br>reverse	GCT CCG TGC CTT TAT CCT G<br>ACG GTG TCC ACC ACA GGT
PTPRZ1	forward<br>reverse	CAT GCC TAT GTT AAT GCA CTC C<br>GCT GTA TAT TTG ACT GGC TCA GG

Supplementary Table 4 Primer sequences
