# Supplementary material for: Region and cell-type resolved quantitative proteomic map of the human heart
Source: Nat Commun. 2017 Nov 13;8:1469. doi: 10.1038/s41467-017-01747-2 (PMC5684139; doi:10.1038/s41467-017-01747-2)
Supplement: Supplementary file 2 — Description of Additional Supplementary Files [file 41467_2017_1747_MOESM2_ESM.pdf]

Description of Additional Supplementary Files

File Name: Supplementary Data 1
Description: Proteins differentially expressed across the heart regions

File Name: Supplementary Data 2
Description: Differentially expressed proteins in the three heart areas

File Name: Supplementary Data 3
Description: Cell volume, copy numbers across all cardiac samples and contribution of each organelle to cellular protein mass

File Name: Supplementary Data 4
Description: Proteins that are found in the top 75% most abundant proteins

File Name: Supplementary Data 5
Description: Differentially expressed proteins in the ventricular versus atrial region

File Name: Supplementary Data 6
Description: Quantified sarcomeric protein isoforms

File Name: Supplementary Data 7
Description: Proteins displaying at least two-fold higher expression in CFs, AFs, ECs, or SMCs

File Name: Supplementary Data 8
Description: Cell membrane receptors displaying at least two-fold higher expression in CFs compared to AFs, ECs, and SMCs

File Name: Supplementary Data 9
Description: Differentially expressed proteins in AFib patients compared to healthy controls
